# Dynamics of Population Activity in Rat Sensory Cortex: Network Correlations Predict Anatomical Arrangement and Information Content

**DOI:** 10.3389/fncir.2016.00049

**Published:** 2016-07-06

**Authors:** Mohammad Mahdi Sabri, Mehdi Adibi, Ehsan Arabzadeh

**Affiliations:** ^1^School of Cognitive Sciences, Institute for Research in Fundamental Sciences (IPM)Tehran, Iran; ^2^Eccles Institute of Neuroscience, John Curtin School of Medical Research, The Australian National UniversityCanberra, ACT, Australia; ^3^Australian Research Council Centre of Excellence for Integrative Brain Function, The Australian National University NodeCanberra, ACT, Australia; ^4^School of Psychology, University of New South WalesSydney, NSW, Australia

**Keywords:** sensory coding, vibrissal, barrel cortex, noise correlation, signal correlation

## Abstract

To study the spatiotemporal dynamics of neural activity in a cortical population, we implanted a 10 × 10 microelectrode array in the vibrissal cortex of urethane-anesthetized rats. We recorded spontaneous neuronal activity as well as activity evoked in response to sustained and brief sensory stimulation. To quantify the temporal dynamics of activity, we computed the probability distribution function (PDF) of spiking on one electrode given the observation of a spike on another. The spike-triggered PDFs quantified the strength, temporal delay, and temporal precision of correlated activity across electrodes. Nearby cells showed higher levels of correlation at short delays, whereas distant cells showed lower levels of correlation, which tended to occur at longer delays. We found that functional space built based on the strength of pairwise correlations predicted the anatomical arrangement of electrodes. Moreover, the correlation profile of electrode pairs during spontaneous activity predicted the “signal” and “noise” correlations during sensory stimulation. Finally, mutual information analyses revealed that neurons with stronger correlations to the network during spontaneous activity, conveyed higher information about the sensory stimuli in their evoked response. Given the 400-μm-distance between adjacent electrodes, our functional quantifications unravel the spatiotemporal dynamics of activity among nearby and distant cortical columns.

## Introduction

Since the classic works by Edgar D. Adrian (Adrian, 1926; Adrian and Zotterman, 1926), continuing progress has been made in understanding how single neurons represent the external world (Parker and Newsome, 1998). With recent advances in array recording and imaging techniques, a body of research has focused on sensory processing at the level of neuronal populations (Panzeri et al., 2015). The population analyses have revealed that cortical neurons fire in a correlated manner (Averbeck et al., 2006; Cohen and Kohn, 2011) and exhibit systematic temporal and spatial structures in their collective activity (Ohki et al., 2005; Miller et al., 2014; Okun et al., 2015). A cortical neuron receives input through a large number of synapses from thousands of other cells (Nicholls et al., 2012). It is thus critical to understand the temporal and spatial structure of activity within cortical populations as such structures shape the way individual neurons process sensory information. A body of work has investigated the functional connectivity in cortical populations, and how this relates to the underlying spatial organization of the network (Cohen and Newsome, 2008; Rothschild et al., 2010; Deco et al., 2011). Specifically, the local structure of connections as inferred by the spontaneous population activity was found to determine the evoked response of neurons to external stimuli (Tsodyks et al., 1999). Collectively, these studies indicate that the short-range and long-range interactions among cortical neurons directly impact the capacity of the population to transmit sensory information.

To gain a quantitative understanding of the spatiotemporal dynamics of cortical networks, here, we used a 10 × 10 array of microelectrodes to sample the ongoing population activity of cortical ensembles. In particular, we investigated the following questions: What is the temporal relationship between the spiking activity of cortical neurons? How does this temporal relationship change with distance between neurons? Do the correlation profiles during spontaneous activity predict the “noise” and “signal” correlations in response to sensory stimulation? How does the strength of correlation between a neuron and the rest of the population affect the neuron's capacity to encode the sensory information?

The rat vibrissal somatosensory cortex (vS1) presents a cortical organization suitable for this study. The vibrissal system is anatomically well-characterized (Petersen, 2007; Feldmeyer, 2012; Feldmeyer et al., 2013) and provides an example of *expert* sensory processing (Diamond and Arabzadeh, 2013). The vS1 cortex is arranged in a topographic map of histologically and physiologically distinct clusters of neurons known as barrels (Woolsey and Van der Loos, 1970; Welker, 1971). The dimensions of the 10 × 10 recording array match the columnar organization of this area of cortex (Arabzadeh et al., 2003), and thus can be employed to reveal the spatiotemporal dynamics of activity across adjacent and distant cortical columns.

## Material and methods

### Surgery and neuronal recording

All experiments were conducted in accordance with international guidelines and were approved by the Animal Experimentation Ethics Committee of the Australian National University. Adult male Wistar rats (*n* = 3) were anesthetized by urethane (1.5 g/kg body weight) and were placed on servo-controlled heating blanket (Harvard instruments) to maintain a steady body temperature near 37 degrees Celsius. The skull was fixed in a custom-built stereotaxic frame and a 7 × 7-mm craniotomy was made to expose the left somatosensory cortex (center of craniotomy: 2-mm posterior and 6-mm lateral to bregma). An array of 10 × 10 electrodes (electrode length: 1500 μm; tip-to-tip distance: 400 μm, Utah) was placed above the somatosensory cortex based on vascular landmarks and the stereotaxic coordinates (Paxinos and Watson, 1986). The array was then inserted into the cortex using a pneumatic inserter (Blackrock) that was set to implant electrodes 1000 μm into the cortex. The dura was left intact.

Signals from 96 electrodes (4 electrodes were not connected) were simultaneously amplified, filtered (250–5000 Hz) and were continuously recorded onto disk at a sampling rate of 30 kHz (Blackrock microsystems Inc., Utah). We then measured the range of the filtered signal on each electrode and applied a threshold of –4.5 standard deviation to detect events. A wavelet-based algorithm (Quiroga et al., 2004) was used to cluster spike shapes. For each electrode all spikes were then pooled to construct the multiunit activity (MUA).

**Figure 1:**
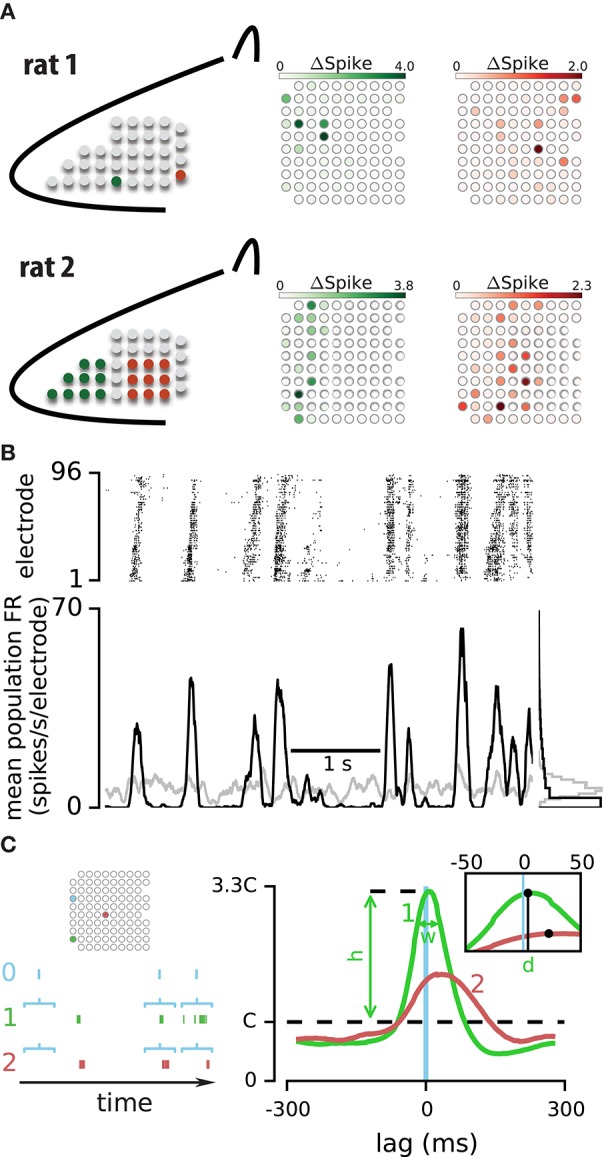
**Strength and temporal profile of correlated activity between electrode pairs**. **(A)** To functionally identify the position of the array relative to the vibrissal cortex, a train of deflections was applied either to individual vibrissae (E4 and delta, upper panel) or to a small number of vibrissae (rostral and caudal, lower panel). The response of electrodes are highlighted with their corresponding color in rat 1 (upper panel) and rat 2 (lower panel). **(B)** Upper panel: the spiking activity simultaneously recorded on 96 electrodes during 5 s of an example episode. Lower panel: mean firing rate across all electrodes for the recorded (in black) and shuffled data (in gray). The histograms to the right compare the distribution of population spiking between real and shuffled data in this episode. **(C)** Left panel: an epoch of spiking activity across three example electrodes. Horizontal brackets illustrate the windows of activity on electrodes “1” and “2” which are triggered by spikes on electrode “0” Right panel: probability distribution function (PDF) of spiking on electrodes “1” (green line) and “2” (red line) relative to spikes on the triggering electrode “0” (blue vertical line). Three parameters; *h, d*, and *w* summarize the correlation profile for each pair of electrodes.

**Figure 2:**
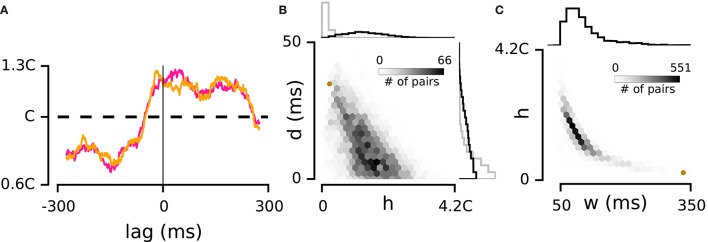
**Interdependence of correlation strength and delay. (A)** Violet and orange curves illustrate two estimations of the spike triggered PDF -calculated with the even and odd spikes on the triggering electrode- for a weakly correlated example pair marked with the brown dot in panels **(B,C)**. **(B)** Main panel: joint distribution of *h* and *d* for all pairs of electrodes in the example episode. For every pair, the absolute value of *d* is shown. The histograms to the top and right compare the distribution of *h* and *d* values between real and shuffled data in this episode. **(C)** Main panel: joint distribution of *h* and *w* across electrode pairs in the example episode. The histogram to the top shows the distribution of *w*.

**Figure 3:**
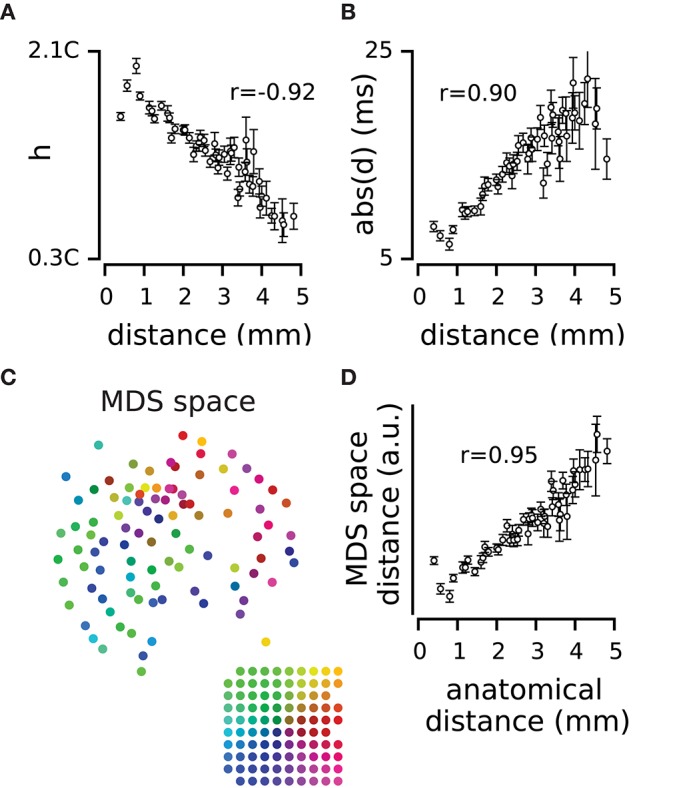
**Spatial dynamics of pairwise correlations. (A)** The mean and s.e.m of strength of correlation, *h*, across electrode pairs at each distance. **(B)** The mean and s.e.m of temporal delay, *d*, across electrode pairs at each distance. **(C)** The position of electrodes in the functional space built based on the *h* values. The functional space is reduced to two dimensions with multi-dimensional scaling (MDS). Colors of electrodes were assigned based on their spatial position as shown in the inset array. **(D)** The mean and s.e.m of distances in the 2-dimensional functional space, at each anatomical distance.

**Figure 4:**
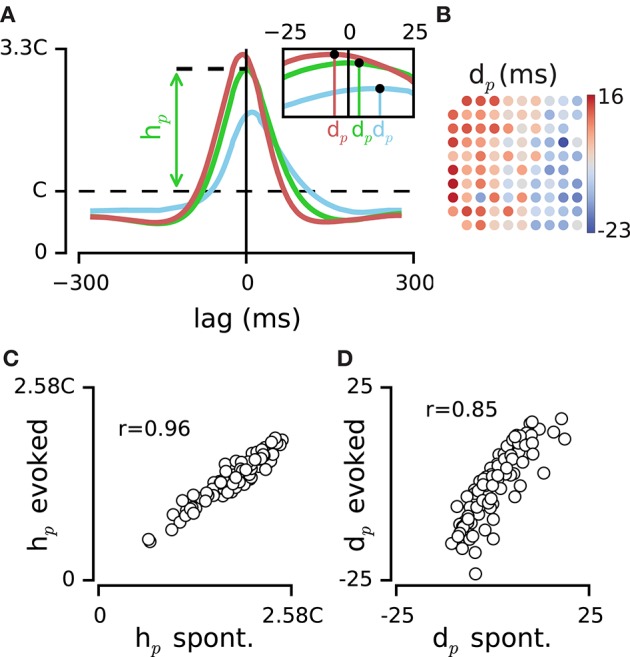
**Dynamics of activity at the population level. (A)** PDF of population activity triggered by spikes on the three example electrodes (electrodes #0, #1, and #2 from Figure 1C). The peak of this PDF relative to chance level (C), denoted by *h*_*p*_, quantifies the dependence of each electrode to the pooled activity of population. Inset figure indicates the median of each PDF, *d*_*p*_, which estimates the delay of each electrode relative to the population spiking at all other electrodes. **(B)** Map of electrodes in the example episode color coded by their *d*_*p*_ value. The layout corresponds to the implanted array in rat 1 (see the functional map in Figure 1A). **(C)** Comparison of *h*_*p*_ values between two example episodes of spontaneous activity and sustained stimulation. Every circle represents one electrode. **(D)** Comparison of *d*_*p*_ values between the same episodes of spontaneous activity and sustained stimulation as in panel **(C)**.

**Figure 5:**
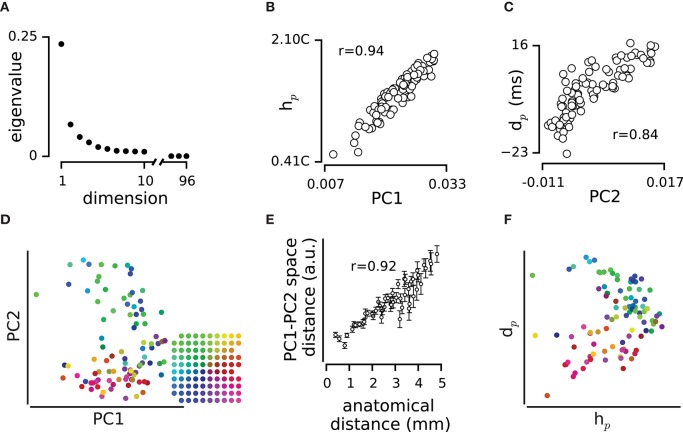
**The relation between the population correlated activity and the pairwise correlated activity. (A)** Eigenvalues resulted from eigendecomposition analysis on matrix of *h* values in the example episode. **(B)** Every circle represents the weight of each electrode in PC1 and its *h*_*p*_ value. **(C)** Every circle represents the weight of each electrode in PC2 and its *d*_*p*_ value. **(D)**, The position of electrodes in a two dimensional space built based on PC1 and PC2 of *h* matrix. Colors of electrodes were assigned based on their spatial position as shown in the inset array. **(E)** The mean and s.e.m of distances in the 2-dimensional PC1-PC2 space, at each anatomical distance. **(F)** The position of electrodes in a 2-dimensional space built based on *h*_*p*_ and *d*_*p*_. Electrodes are color coded as in panel **(D)**.

**Figure 6:**
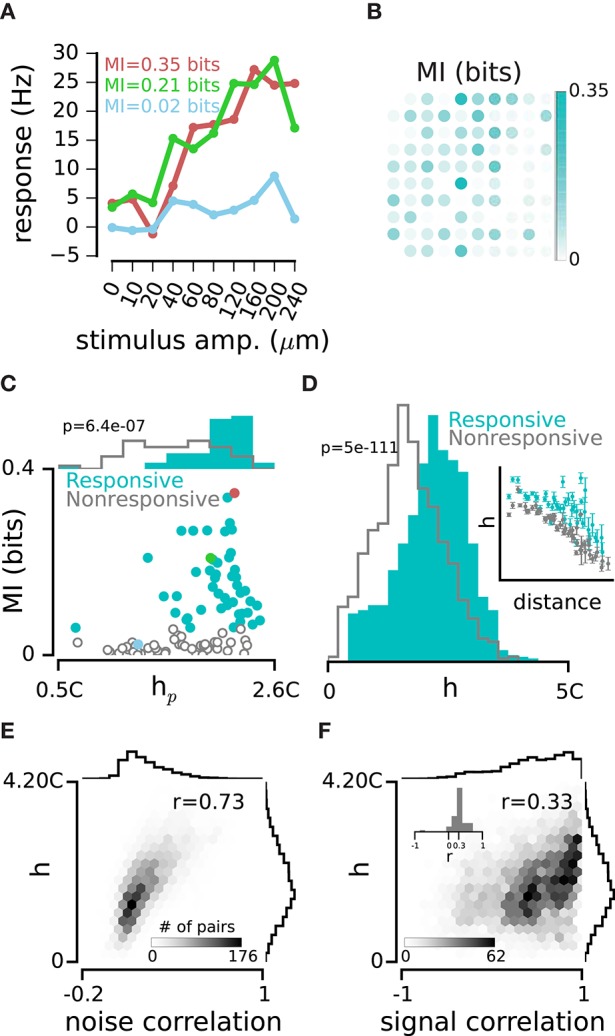
**Network dependency and sensory coding. (A)** Response functions of the three example electrodes. Colors indicate the same example electrodes as in Figures 1C, 4A. **(B)** Mutual Information (MI) between spike counts on each electrode and the stimulus set in Rat 1. Spike counts were calculated in a 0-100 ms window post stimulus onset. **(C)** Every dot represents one electrode. Electrodes are divided into *Responsive* (cyan) and *Nonresponsive* (gray) groups separated by the median MI value. Top histograms compare *h*_*p*_ distributions for the two groups of electrodes. **(D)** Normalized distribution of *h* values for *Responsive* pairs (where both electrodes in a pair were from the *Responsive category*; cyan) and *Nonresponsive* pairs (where both electrodes in a pair were from the *Nonresponsive* category; gray). Inset is similar to Figure 3A, only plotting *h* separately for the *Responsive* (cyan) and *Nonresponsive* pairs (gray). **(E)** *h* values are measured in an example episode of spontaneous activity. The histograms show the distributions of *h* values and noise correlations. **(F)** Same as panel E, but for signal correlation. Inset histogram: every *r* value is calculated within groups of equidistant pairs of electrodes. The distribution of *r* values is positive with a mean of 0.3 indicating that the positive correlation between *h* and signal correlation is present at all distances.

We applied brief periods of single whisker stimulation to obtain the functional mapping of the electrodes (Figure 1A). For the main recording session, episodes of spontaneous activity were interleaved with episodes of sustained stimulation of the whole-whisker-pad (Figures 2–**5**). Finally to measure the information content of each unit, we stimulated the whole-whisker-pad with brief (25-ms) deflections at different amplitudes (**Figure 6A**).

Data collected from one rat included only recording of spontaneous activity. In two other rats, three episodes of spontaneous activity were interleaved with two episodes of sustained sensory stimulation. Each episode had a minimum duration of 12 min. Sustained stimulation comprised a continuous sine wave (frequency: 40 Hz; amplitude: 80 or 240 μm) applied to the whole vibrissal pad using a mesh driven by a piezo-electric wafer. Electrodes with low signal quality in the first recording episode were excluded from the analyses. This resulted in 96, 62, and 52 active electrodes in rats 1, 2, and 3. One episode of recording in rat 2 was excluded from analyses because of the presence of electrical noise. To quantify the responsiveness of electrodes, each recording session also included an episode of intermittent stimulation. Here, brief bipolar deflections (25-ms) were applied to the vibrissal pad interleaved with 1 s inter-trial intervals. A range of amplitudes was applied in a pseudorandom order for 100 repetitions per amplitude. These included 10 amplitudes in rat 1 (0, 10, 20, 40, 60, 80, 120, 160, 200, 240 m) and 11 amplitudes in rat 2 (0, 5, 10, 20, 40, 60, 80, 120, 160, 200, 240 m). At the end of the recording sessions, specific vibrissae were stimulated (either individually or in a small number) to identify the functional map of the array relative to the vS1 cortex (Figure 1A).

### Data analyses

Data analyses were performed in the IPython Notebook environment (Pérez and Granger, 2007) using the Python kernel (www.python.org). Figure 1C shows the calculation procedure for the spike-triggered probability density function (PDF). Horizontal brackets indicate example windows of activity on electrodes #1 and #2, which were aligned by spikes on electrode #0. We computed the probability distribution of activity on electrodes #1 and #2 at each time point conditioned to the spikes on electrode #0. Right panel in Figure 1C plots the probability distribution of spiking on electrode #1 (green) and #2 (red) conditional on spikes observed on electrode #0 after convolving the traces with a 50 ms rectangular window. This spike-triggered PDF is mathematically the same as the normalized cross correlogram. When activities on two electrodes are uncorrelated, the spike-triggered PDF will exhibit a uniform distribution (i.e., the dashed line in Figure 1C, right panel) indicating that the observation of a spike on one electrode does not alter the probability of spiking on the other electrode. We defined the height of this uniform distribution as the chance level (C) and used it to characterize the strength and profile of spike-triggered PDFs.

To build the functional dissimilarity matrix across electrode pairs, we used the following equation:

(1) $$\begin{array}{l} f_{ij}=1-\frac{h_{ij}}{\sqrt{h_{ii}\times h_{jj}}} \end{array}$$

where *f*_*ij*_ represents the functional distance between electrodes *i* and *j, h*_*ij*_ represents the peak of spike-triggered PDF of electrode *i* against electrode *j*, relative to the chance level (C).

We divided each recording episode into 40-s segments. These segments were then randomly shuffled individually for each electrode. This procedure preserves the local temporal profile of activity within each electrode and diminishes the correlation of activity across electrodes.

To calculate the noise correlation of responses during intermittent stimulation episodes, neural responses were defined as the spike count (0–100 ms post stimulation onset) on each recording electrode. The same spike count was used to calculate the mutual information (Ince et al., 2010) between neural responses and sensory stimuli (**Figure 6**). The neuronal response function to sensory stimulation was estimated during intermittent stimulation episodes (**Figure 6A**). Neuronal response was defined as the change in firing rate in the 100 ms window post-stimulus onset relative to the baseline (50 ms window before stimulus onset).

For each of the main findings, the statistical significance is examined and reported separately for individual recording episodes (*n* = 9). Figures 2–**6** show the observations in an example episode while the text quantifies how the findings generalize across episodes by providing the range of correlation coefficients and *p*-values.

## Results

To study the spatiotemporal dynamics of neural activity in a cortical population, we implanted a 10 × 10 array of microelectrodes in the vibrissal field of the somatosensory cortex of urethane-anesthetized rats (Figure 1A). We recorded spontaneous activity and activity evoked in response to sustained and transient sensory stimulation. Figure 1B illustrates 5 s of spiking activity across 96 electrodes during an example episode. As visualized in the raster plot, the neuronal population exhibits periods of correlated activation: transient increases in spiking across electrodes interleaved with periods of low activity. Consistent with previous studies (Luczak et al., 2015), instances of high and low activity occurred more frequently than expected by chance (based on comparison to randomly-shuffled sequences, Figure 1B; see Methods). How is the correlated population activity reflected in the temporal structure of spiking between pairs of electrodes?

To quantify the temporal relation of activity across two electrodes, we computed the probability distribution function (PDF) of spiking on one electrode given the observation of a spike on another electrode. This spike-triggered PDF is the same as normalized cross correlogram of two electrodes and provides an easy method to check the consistency of the correlation profiles by using subsets of triggering spikes for PDF estimation (see below). Figure 1C illustrates the probability of spiking on electrodes #1 and #2 relative to the spikes on electrode #0. Three parameters summarize the temporal relations of spiking across two electrodes: (1) The strength of correlation, quantified by *h*: the peak of the spike-triggered PDF relative to the chance level (C, see Methods). (2) The temporal delay in activity across the two electrodes, quantified by *d*: the median of the spike-triggered PDF. (3) The temporal precision of correlated activity, quantified by *w*: defined as the width at ¾ peak height of the spike-triggered PDF. Examining the three parameters *h, d*, and *w* for the two electrode pairs (0–1 and 0–2) revealed specific temporal patterns: the most probable time of spiking on electrode #1 was 7 ms after spikes on electrode #0, whereas the most probable time of spiking on electrode #2 was 23 ms after spikes on electrode #0. Comparison of parameter *h* between the two PDFs indicated that the activity on electrode #0 was more strongly linked to the activity on electrode #1 than electrode #2.

To verify the consistency of these estimates, we calculated the spike-triggered PDF separately with the “odd” and “even” spikes on the triggering electrode (i.e., electrode “0” in Figure 1C). We compared *h* values computed with these two non-overlapping sets of spikes (odd and even) for all pairs of electrodes in every recording episode. The correlation coefficient of the two sets of *h* values was 0.99. Similarly, performing this consistency test on parameters *d* and *w* revealed correlation coefficients of 0.96 and 0.90, indicating that our data set allowed robust estimation of these three parameters. Figure 2A shows an example of the two PDFs estimated based on the odd and even spikes. Although this pair exhibited a relatively weak correlation, the overlap between the two traces indicates consistent estimation of the three parameters. In the next section we explore the significance of these parameters and their interaction.

### Parameters *d, h*, and *w* are interdependent

To reveal the dynamics in the space of pairwise correlations, we investigated the individual and joint distributions of *h, d*, and *w*, and compared them with the distributions obtained from shuffled data.

For the example episode in Figure 2B, the average *h* was 1.4C which was reduced to 0.2C by shuffling. Across all episodes, the *h* values ranged from 0.07C to 6.41C (median: 1.28C; interquartile range: 0.85C–1.82C, *n* = 9). Data shuffling (see Methods) reduced the range of *h* values to a median of 0.19C (interquartile range: 0.11C–0.34C, *n* = 9). The distribution of *d* values (delay between the activity of electrode pairs) covered a broader range compared to the shuffled data (*p* = 2e–60; Levene test for equivalence of variance, see the distribution of *d* for the real and shuffled data in Figure 2B for the example episode). Next we examined the relationship between the strength of correlation (*h*) and the temporal delay (*d*). Strong correlations occurred when delay between electrode pairs was short, and correlations decreased as delays got longer (*r* = –0.4 ± 0.12, mean ± sd across recording episodes, *n* = 9, all *p* < 0.001, see Figure 2B for the example episode).

We then quantified the distribution of *w* and its joint distribution with *h* (e.g., Figure 2C). This analysis was limited to electrode pairs for which the *h* and/or *d* were above 95% of the shuffled distribution (91% of electrode pairs passed this criteria). As expected, strongly correlated pairs (high *h*) showed low *w* and conversely high values of *w* corresponded to weak correlations.

### Temporal dynamics change with distance

To investigate how distance between two electrodes might determine their profile of correlation, we studied the spatial dynamics of *h, d*, and *w*. Overall, by increasing distance the strength of correlation, *h*, declined (*r* = –0.87 ± 0.06, mean ± sd across recording episodes, *n* = 9, all *p* < 0.001; e.g., Figure 3A), the delays got longer (*r* = 0.42 ± 0.39, mean ± sd, *n* = 9, correlation coefficient was significant in 6 episodes, *p* < 0.001; e.g., Figure 3B) and the width increased (*r* = 0.69 ± 0.16, mean ± sd, *n* = 9, all *p* < 0.001). However, at low distances (< 800 μm), all three parameters showed the opposite trend: a rise in *h* (*r* > 0.95, across all episodes, *n* = 9), and a drop in *d* (*r* < –0.91, across all episodes, *n* = 9) and *w* (*r* < –0.83, across all episodes, *n* = 9). Across recording episodes, we observed a systematic spatial profile of correlations: the strongest correlation, the minimum delay and the minimum temporal dispersion occurred when the distance between two electrodes was 800 micrometers.

To what extent does the functional connectivity between two electrodes determine their physical position on the cortical surface? We used *h* as the measure of similarity between two electrodes and built a dissimilarity matrix that captures the functional distance between every pair of electrodes (see Equation 1 in Methods). We applied multidimensional scaling (MDS; Kruskal, 1964; Pedregosa et al., 2012) to the dissimilarity matrix and estimated the functional space of electrodes with different dimensions. Figure 3C visualizes the functional position of electrodes in a two-dimensional space color-coded to their anatomical position. The functional position of electrodes in the MDS space maintains their physical arrangement on the cortical surface. Figure 3D further verifies the correspondence between the two maps in terms of the correlation between the anatomical distances and the functional distances in the two dimensional MDS space (*r* = 0.95 for the example episode). This correlation was reproduced across all recording episodes (*r* = 0.90 ± 0.05, mean ± sd across recording episodes, *n* = 9, all *p* < 0.001). We examined this correlation when representing the functional space in different dimensions; the most prominent change in functional space (relative to anatomical space) occurred in transition from one to two-dimensional space (57 ± 31%, mean ± sd across recording episodes, *n* = 9). For dimensions higher than two, changes in the relation of functional space and anatomical space remained relatively small (less than 5 ± 3%, mean ± sd, *n* = 9).

### Dynamics of activity at the population level

How are the spikes of individual neurons coordinated relative to the activity of the whole population? How does the temporal arrangement of spikes from individual neurons depend on their spatial arrangement in the network? To address these questions, we quantified for every electrode, the probability distribution of population activity of the rest of the network (pooled spiking of all other electrodes) around spike times of that electrode. Similar to the pairwise quantifications, the strength of correlation between the electrode and the rest of population, denoted by *h*_*p*_, was measured as the peak of the spike-triggered PDF relative to the chance level (Figure 4A). Across recording episodes, *h*_*p*_ values ranged from 0.42C to 2.46C (median: 1.33C; interquartile range: 1.06C–1.67C, *n* = 9), demonstrating moderate to high level of temporal correlation with the rest of the population. The *h*_*p*_ values remained highly consistent across episodes of spontaneous activity and sustained stimulation (*r* = 0.94 ± 0.03, mean ± sd across all episode pairs, *n* = 10, all *p* < 0.001, e.g., Figure 4C).

For each electrode, the median of the distribution denoted by population delay, *d*_*p*_, quantifies the temporal delay of spiking on that electrode relative to the population activity (Figure 4A inset). The values of *d*_*p*_ for episodes of spontaneous activity were highly correlated with those for the sustained stimulation (*r* = 0.82 ± 0.07, mean ± sd, across all episode pairs, *n* = 10, all *p* < 0.001; e.g., Figure 4D). This indicated that the sequence of activation among electrodes was highly preserved between the two conditions of spontaneous and sensory evoked activity. The *d*_*p*_ changed systematically from positive (leading the population) to negative (lagged relative to the population) values and this clustering was most evident across the rows of the recording array, which approximately corresponded to transition from rostral to caudal whiskers (see Figure 4B for the example episode). We divided the electrodes into two groups based on the median of *d*_*p*_ values across all electrodes: leading group and delayed group. Anatomical distance of the two groups was 1.23 ± 0.47 mm (mean ± sd) across all episodes (*n* = 9). Shuffling the position of electrodes reduced this distance to 0.32 ± 0.18 mm (mean ± sd, *n* = 9). Such systematic relationship with the position of electrodes was not observed in the values of *h*_*p*_ (data not shown).

Here, we quantified the temporal relations of spiking for pairs of electrodes (in terms of *h* and *d*, Figures 1–3), and at the population level (in terms of *h*_*p*_ and *d*_*p*_, Figure 4). The measures *h* and *d* are bounded to pairs of neurons, and do not explicitly depict the spatiotemporal patterns among the whole population as quantified by *d*_*p*_ and *h*_*p*_. To generalize the analysis from the pairwise level to the population level, we employed a method based on the eigendecomposition of the correlation matrix *h*. This is identical to principal component analysis on the activity of the population: for a population of *N* neurons, the eigendecomposition results in a set of *N* orthonormal vectors (eigenvectors) and *N* associated eigenvalues each of which identifies the contribution of its eigenvector to represent the functional space (matrix *h*) in terms of uncorrelated principal components. We observed that eigenvalues declined exponentially in their value (e.g., Figure 5A). The first two eigenvectors captured 47 ± 4% (mean ± sd, across all episodes, *n* = 9) of the diversity in the functional space, with the first eigenvector alone representing 36 ± 4% (mean ± sd, across all episodes, *n* = 9). Consistent with our previous results (Adibi et al., 2013, 2014), the first eigenvalue and its corresponding eigenvector characterized the overall strength of the correlations across the population: the first principal component (PC1) captured the strength of correlation *h*_*p*_ (*r* = 0.96 ± 0.02, mean ± sd across recording episodes, *n* = 9, all *p* < 0.001; e.g., Figure 5B). The second principal component (PC2), however, represented *d*_*p*_ (*r* = 0.67 ± 0.11, mean ± sd across recording episodes, *n* = 9, all *p* < 0.001; e.g., Figure 5C). Thus, the pairwise correlations (functional connectivity map, *h*) can be explained in terms of PC1 representing the overall strength of correlations with population (or *h*_*p*_) and PC2 representing the order of neurons in the sequence of population activity (*d*_*p*_).

The dimensionally-reduced functional space of PC1 and PC2 values form a flat functional map of electrodes (e.g., Figure 5D). PC1 and PC2 were driven from pairwise correlation values in *h* which are a function of the anatomical distance (Figure 3). Additionally, PC2 was correlated with *d*_*p*_ which in turn correlated with the relative position of electrodes on cortex (Figure 4B). Thus, the two dimensional functional map of electrodes resembles the physical map of electrodes on the array (Figure 5D). This motif was verified by high level of correlation between pairwise distances in the functional map and the anatomical map (*r* = 0.74 ± 0.23, mean ± sd across recording episodes, *n* = 9, correlation coefficient was significant in 8 episodes, *p* < 0.001, e.g., Figure 5E). Figure 5F illustrates how a two dimensional space built based on *h*_*p*_ and *d*_*p*_ values replicated the anatomical position of electrodes.

### Network dependency and sensory coding

Does the strength of correlation with the rest of the network determine a neuron's capacity to encode the sensory input? Figure 4A quantified the strength of correlation between single electrodes and the population activity pooled across all other electrodes, as denoted by *h*_*p*_. Here we use the quantity *h*_*p*_ as a measure of network dependence. Figure 6A shows the responses of the three example electrodes to the range of vibration amplitudes applied to the whisker pad during the intermittent stimulation episode. To quantify how well stimulus amplitude is encoded by spike trains recorded on each electrode, we computed the mutual information between stimuli and neuronal responses. Mutual information (Shannon, 1948) quantifies the amount of information that neuronal responses provide about the sensory stimuli on a trial-by-trial basis (Cover and Thomas, 1991). After correcting for sampling bias (Ince et al., 2010), the MI values ranged from 0 to 0.74 bits (across electrodes and recording sessions, median MI: 0.06 bits, interquartile range: 0.01–0.17 bits, see Figure 6B for the rat 1). Although electrodes #1 and #2 had similar response functions (green and red curves in Figure 6A), their MI values were 0.21 bits and 0.35 bits, respectively. This shows that trial-to-trial variability was lower in electrode #2 compared to electrode #1. We observed a modest but significant correlation between network dependence, *h*_*p*_, and information content of electrodes, MI (*r* = 0.47 ± 0.04, mean ± sd across recording episodes, *n* = 9, all *p* < 0.001). We then divided the electrodes into two groups of “responsive” and “nonresponsive” based on the median of the MI distribution. Responsive electrodes showed a systematically higher *h*_*p*_ than nonresponsive electrodes (*p* < 0.001, across all episodes, *n* = 9, e.g., Figure 6C). Consistent with this observation, the pairwise *h* among responsive pairs was higher than that among nonresponsive pairs (*p* < 0.001 across recording episodes, *n* = 9, e.g., Figure 6D). For both groups, *h* values declined with distance (among responsive pairs *r* = −0.38 ± 0.28, mean ± sd across recording episodes, *n* = 9, correlation coefficient was significant in 5 episodes, *p* < 0.001 and among nonresponsive pairs *r* = −0.84 ± 0.08, mean ± sd across recording episodes, *n* = 9, all *p* < 0.001; inset in Figure 6D). However, at all distances, higher network dependence (higher *h*) corresponded to higher sensory coding capacities.

Previous analysis demonstrated that neurons that were strongly coupled to the network during their spontaneous activity also exhibited a higher degree of responsivity to sensory stimulation. Here we asked whether the fine pairwise correlation profile during spontaneous activity could also predict the correlation of sensory evoked responses. We examined two types of correlation between electrodes, the “signal” and “noise” correlations. Noise correlation quantifies the trial-to-trial covariability in response to the same stimulus, whereas signal correlation quantifies the correlation of response functions across stimuli (Figure 6A, see Methods). As expected, the strength of correlation, *h*, was highly predictive of noise correlation between two electrodes (*r* = 0.75 ± 0.03, mean ± sd across recording episodes, *n* = 9, all *p* < 0.001, e.g., Figure 6E). We also found that *h* exhibited a weak, but robust relation to signal correlation (*r* = 0.29 ± 0.06, mean ± sd, across recording episodes, *n* = 9, all *p* < 0.001, e.g., Figure 6F). To check if the distance of electrodes could explain the relation of *h* and signal correlation, we examined this relation in different groups of equi-distant pairs of electrodes; the distribution of correlation coefficients confirmed the consistency of this relation across all distances (inset in Figure 6F).

## Discussion

We investigated the spatiotemporal dynamics of neural activity in a population of neurons recorded from the vibrissal somatosensory cortex. The spike-triggered probability distribution functions illustrated the correlation profile of electrode pairs in terms of the strength (*h*), temporal delay (*d*), and temporal precision (*w*) of correlated activity. The parameters, *h, d*, and *w* were reliably estimated within each recording episode, and maintained their value and relationship to each other across episodes of spontaneous activity and sustained sensory stimulation. Highly correlated pairs tended to exhibit small delays and as the correlation strength decreased the delay increased. The parameters *h, d*, and *w* showed a systematic relation with distance. Neurons that were strongly coupled to the network during their spontaneous activity conveyed higher amounts of information about the sensory stimulus. Finally, the fine correlation profile of electrode pairs during spontaneous activity could predict noise and signal correlations during sensory stimulation.

Rodents use their vibrissae to navigate the environment and collect information about various aspects of the contacted object such as its size (Brecht et al., 1997), position (Knutsen et al., 2006; Knutsen and Ahissar, 2009; O'Connor et al., 2010), or surface texture (Diamond et al., 2008; Morita et al., 2011). In the vibrissal area of the primary somatosensory cortex, neurons generate reliable representations of these aspects by encoding the kinematics of whisker movements such as high velocity events that occur during contact with rough surfaces (Arabzadeh et al., 2005; Wolfe et al., 2008). The overall rate of action potentials of a single neuron or a neuronal population carries information about the velocity of vibrations applied to the whiskers (Arabzadeh et al., 2004) and behavioral experiments indicate that rats are sensitive to the mean velocity of their whiskers (Gerdjikov et al., 2010; Adibi and Arabzadeh, 2011; Adibi et al., 2012). Furthermore, precise timing of action potentials both within and across individual neurons may carry extra information about sensory events (Panzeri et al., 2002; Arabzadeh et al., 2006; Montemurro et al., 2007). Correlations of activity across individual neurons within the population are found to affect the transmission of sensory information (Adibi et al., 2013, 2014) as has also been found in other modalities (Cohen and Kohn, 2011; Ponce-Alvarez et al., 2013). It is however, not clear to what extent these various coding schemes contribute to the representation of the sensory environment, and are “read out” by downstream neurons to influence behavior (Zuo et al., 2015). When decoding the population activity, the relative spatial position of neurons, their connectivity and their cellular and synaptic properties is expected to affect the information content carried in the population response (Miller et al., 2014; Okun et al., 2015). Characterizing the spatial and temporal dynamics of the neuronal population activity can thus identify the computational constraints on the population activity, and shed light on the underlying mechanisms of cortical information processing.

The pattern of population spiking activity in a local circuit covers a continuum in which two extremes define the state of cortical activity: the synchronized and desynchronized states. In the synchronized state the local population shows brief (50–100 ms) periods of synchronized activation separated by periods of inactivity (Harris and Thiele, 2011). These packets of population activity in the synchronized state, accompanied by periods of silence, impose a high level of correlated activity between adjacent neurons (Mochol et al., 2015; Scholvinck et al., 2015). During the desynchronized state these segregated packets do not exist and neurons exhibit lower levels of correlated activity and thus an enhanced stimulus representation (Marguet and Harris, 2011; Zagha et al., 2013; Pachitariu et al., 2015). Recent evidence suggests that different states could simultaneously exist in different cortical regions (Vyazovskiy et al., 2011; Zagha et al., 2013). It is not clear how the cortical state affects the degree of network dependence of cells. Our recordings here were performed under urethane anesthesia, and thus mainly reflected the spatiotemporal dynamics of activity in the synchronized state. Future experiments could apply similar analyses during waking and examine how various cortical states influence the dynamics of population activity.

Previous recordings with multielectrode arrays identified temporal dynamics of local population of finely separated neurons that were broadly consistent with our findings here (Luczak et al., 2007, 2013). At such fine spatial scales, the relative timing of neurons' spiking activity exhibited a sequential temporal structure with systematic patterns that were also preserved during various states of the network (Luczak et al., 2007, 2013; but see Pachitariu et al., 2015). Here we investigated the spatiotemporal dynamics of activity among distant neurons. The 400 μm distance between adjacent electrodes in the Utah array is similar to the diameter of cortical columns which are considered the computational modules in primary sensory cortex and other cortical areas (Mountcastle, 1957; Hubel et al., 1977; Buxhoeveden and Casanova, 2002; Burgalossi and Brecht, 2014). Our findings therefore characterize the spatiotemporal dynamics of activity across adjacent and distant cortical columns.

We found that the strength of correlation decreased with distance. This finding is consistent with the general observation that the correlation strength declines with distance in different areas of cortex (Smith and Kohn, 2008; Rothschild et al., 2010; Solomon et al., 2014). However, we also observed an initial rise in correlation at short distances (up to 800 μm) which was followed by a steady drop in correlation (Figure 3A). Consistent with this observation, Ajima and Tanaka (2006) reported that in L2/3 vibrissal cortex, fast inhibitory connections decline more rapidly with distance than do the excitatory connections. The original rise can also be due to the stronger connections between adjacent barrels compared to the barrel and septum connectivity, observed in the supragranular layers (Alloway, 2008).

A powerful method for determining population dynamics is eigendecomposition analysis of the neuronal space. Reyes-Puerta and colleagues applied this method to population activity in the vS1 recorded with multi-shank linear arrays (Reyes-Puerta et al., 2015a; see also Adibi et al., 2013, 2014). Their eigendecompostion, performed on the spike count correlation matrix, revealed that a small number of dimensions summarize variances in the space of population activity. Our eigendecomposition analysis, performed on the matrix of *h* values, further confirms these results (Figure 5A). Moreover, we found that the contribution of each unit to the first and second principal components was captured by the unit's *h*_*p*_ and *d*_*p*_ values, respectively, (Figures 5B,C). The orderly arrangement of *d*_*p*_ values across the cortical surface (Figure 4B) suggests a directional flow of activity, and this is consistent with the eigendecomposition analysis of Reyes-Puerta et al. (2015a) demonstrating the propagation of activity along the barrel rows (see also Petersen et al., 2003; Civillico and Contreras, 2012).

We recorded neural population activity across a 3.6 × 3.6 mm^2^ area of the rat cortex. Given the complex three-dimensional architecture of cortical columns (Egger et al., 2012) and the cortical curvature, the recordings could not be attributed to specific columns, layers, and cell types. Therefore, here we have assumed an oversimplified model of the cortex whereby all analyses focused on the spatial distance across electrode tips without including the three-dimensional structure of the cortex and the differences across neurons. To better understand the dynamics of sensory processing, a powerful approach is to identify how a neuron's cell type and excitatory/inhibitory nature determine its network dependence and information content (Reyes-Puerta et al., 2015b). Future experiments could combine population recordings using linear arrays with juxta-cellular recording/labeling (Pinault, 2011) to better establish the network coupling for morphologically identified neurons.

We observed that the fine correlation profile of electrode pairs during spontaneous activity could predict noise correlation as well as signal correlation during sensory stimulation. Furthermore, the strength of correlation between an electrode and the rest of the network determined the amount of information it carried about the sensory input. This is consistent with recent findings by Okun and colleagues who reported that more responsive neurons (to sensory or optogenetics stimulation) were more coupled to the network (Okun et al., 2015). Okun et al. focused on a population of neurons recorded in 600 μm of rat visual cortex. Our recordings covered a 3.6 × 3.6 mm^2^ area, and mainly included multi-unit activity from clusters of neurons in the vS1 cortex (Figure 1A). The similarity of findings suggests that the relation between network coupling and sensory coding capacity may generalize across cortical areas and be present at multiple scales.

## Author contributions

MS participated in the design of the study, analyzed the data, prepared the figures and drafted the manuscript; MA participated in data collection and analysis; EA participated in the design of the study, and data collection, coordinated the study and helped draft the manuscript. All authors modified the manuscript and gave final approval for publication.

## Funding

Supported by an Australian Research Council (ARC) Discovery Project (EA; DP130101364) the ARC Centre of Excellence for Integrative Brain Function (CE140100007). MA supported by a NHMRC CJ Martin Early Career Research Fellowship.

### Conflict of interest statement

The authors declare that the research was conducted in the absence of any commercial or financial relationships that could be construed as a potential conflict of interest.
